# Primary Root Excision Induces ERF071, Which Mediates the Development of Lateral Roots in Makapuno Coconut (*Cocos nucifera*)

**DOI:** 10.3390/plants12010105

**Published:** 2022-12-26

**Authors:** Mya Thuzar, Yonlada Sae-lee, Chatree Saensuk, Mutiara K. Pitaloka, Punyavee Dechkrong, Wanchana Aesomnuk, Vinitchan Ruanjaichon, Samart Wanchana, Siwaret Arikit

**Affiliations:** 1 Rice Science and Innovation Center, Kasetsart University Kamphaeng Saen Campus, Nakhon Pathom 73140, Thailand; 2 Department of Agronomy, Faculty of Agriculture at Kamphaeng Saen, Kasetsart University Kamphaeng Saen Campus, Nakhon Pathom 73140, Thailand; 3 Central Laboratory and Greenhouse Complex, Research and Academic Services Center, Faculty of Agriculture at Kamphaeng Saen, Kasetsart University Kamphaeng Saen Campus, Nakhon Pathom 73140, Thailand; 4 National Center for Genetic Engineering and Biotechnology (BIOTEC), National Science and Technology Development Agency (NSTDA), Pathum Thani 12120, Thailand

**Keywords:** embryo rescue, makapuno coconut, root excision, root development, transcriptome, ethylene responsive factor, *ERF071*, RNA-seq

## Abstract

Coconut (*Cocos nucifera* L.) is widely recognized as one of nature’s most beneficial plants. Makapuno, a special type of coconut with a soft, jelly-like endosperm, is a high-value commercial coconut and an expensive delicacy with a high cost of planting material. The embryo rescue technique is a very useful tool to support mass propagation of makapuno coconut. Nevertheless, transplanting the seedlings is a challenge due to poor root development, which results in the inability of the plant to acclimatize. In this study, primary root excision was used in makapuno to observe the effects of primary root excision on lateral root development. The overall results showed that seedlings with roots excised had a significantly higher number of lateral roots, and shoot length also increased significantly. Using de novo transcriptome assembly and differential gene expression analysis, we identified 512 differentially expressed genes in the excised and intact root samples. *ERF071*, encoding an ethylene-responsive transcription factor, was identified as a highly expressed gene in excised roots compared to intact roots, and was considered a candidate gene associated with lateral root formation induced by root excision in makapuno coconut. This study provides insight into the mechanism and candidate genes involved in the development of lateral roots in coconut, which may be useful for the future breeding and mass propagation of makapuno coconut through tissue culture.

## 1. Introduction

Coconut (*Cocos nucifera* L.), the tree of life, is one of nature’s most useful trees, and is widely known for its uses in oil, food, wood, and fiber. A unique type of coconut, called Makapuno or Maphrao khati in Thai, is characterized by its soft, jelly-like endosperm [1]. Makapuno is a high-valued commercial coconut with distinct sensory attributes, and this type of coconut is an expensive delicacy whose planting material has a high price, especially in Thailand. Despite the high demand from customers, production of makapuno is still limited, so farmers are very interested in growing this variety. However, there is a limitation on the production of seedlings, because this coconut has a unique type of endosperm that cannot naturally support embryo germination [1]. Consequently, makapuno nuts cannot germinate naturally. Therefore, the embryo rescue technique is a very useful tool to support the mass propagation of makapuno coconut.

The first attempt to isolate and culture zygotic embryos from makapuno coconut was made by a group led by Dr. Emerita V. de Guzman from the University of Philippines [2], and it achieved a 50–70% yield of germinating embryos on White’s medium [3]. Their subsequent study on the addition of a high level of sucrose (8%) and IAA (10 mg/L) to White medium showed axillary root production and contributed to the establishment of a desirable shoot–root ratio [2]. Despite an internationally accepted standard protocol for coconut embryo germination being developed, the success rate of the transition from cultured seedlings to plants growing in soil is very low, ranging from 0–50% [4,5]. The main obstacle in tissue culture coconut production is the low success in acclimatization, because many plants do not have functioning roots, or the photosynthetic capacity of the seedlings is low. There are many approaches to improve root induction and acclimatization; one technique with the greatest success (over 95%) is to use a photoautotrophic system [6]. However, this technique is costly and requires a complex system to supply CO_2_-enriched air.

Root production is considered an important contribution to improving seedling quality [6,7]. Recently, we made several attempts to develop an efficient and rapid protocol for makapuno germination focusing on the root system. In one of the preliminary attempts, we tried to excise the primary root of the embryo culture and observed that plants with excised roots grew significantly faster than plants with intact roots. It is well known that cutting plant organs such as stems stimulates the growth of new functional roots and the regeneration of the root system in plants [8,9,10,11,12]. In Arabidopsis, root excision, also known as root pruning, has been reported to lead to root system renewal and increase the number of lateral roots, which is associated with YUC-mediated auxin biosynthesis [13]. Root regeneration after wounding or injury is primarily associated with the auxin biosynthetic pathway in Arabidopsis [14]. Auxin is important for many aspects of root development, including initiation, patterning, and gravitropism, and it is especially important for all stages of lateral root development [15,16,17]. Other hormones, such as ethylene, are also involved in root development [18]. In Arabidopsis, the formation of lateral roots has been reported to be regulated by ethylene [19]. Ethylene biosynthesis could be induced by wounding and ethylene signals through Apetala2/ethylene responsive factors (AP2/ERFs), which have been reported to be involved in stress responses and adventitious and lateral root formation in plants [20]. Interactions between ethylene and auxin have been reported to regulate lateral root initiation and emergence in Arabidopsis [21].

Although the formation of lateral roots and their molecular system is well studied in the model plant Arabidopsis [22] and some other crops [23,24], there are few reports on lateral root development in coconuts. Moreover, the mechanisms underlying the regeneration process after root excision in coconuts are poorly understood. In this study, we aimed to describe the effects of root excision on promoting lateral root initiation and development, and to determine relevant genes associated with lateral root growth induced by primary root excision in coconuts.

## 2. Results

### 2.1. Makapuno Embryo Culture and Investigation of the Impact of Primary-Root Excision on Lateral-Root Development and Shoot Growth

**Figure 1:**
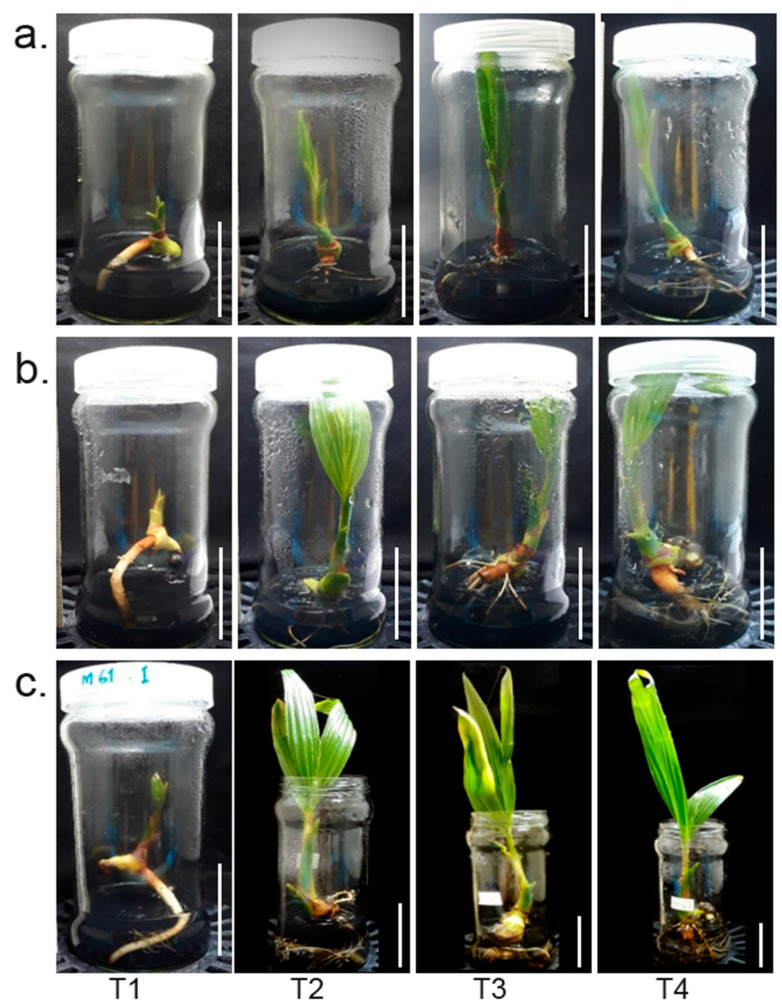
Seedling growth under different root-excision treatments at different timepoints. Embryo cultures of control (intact root: T1) and three root excision treatments, 0.5 cm (T2), 1.0 cm (T3), and 2.0 cm (T4), at three stages: (**a**) 1 month, (**b**) 2 months, and (**c**) 3 months after root excision, respectively. The scale bar represents a length of 5 cm.

Embryos of makapuno coconut cultured in Y3 medium (BA and NAA) were found to germinate within two months with a high germination rate of 80–100%, indicating that the medium used in this experiment functioned well for makapuno embryo rescue. Once the primary root emerged, the embryos were subjected to a root excision experiment with three excision patterns (treatments), i.e., excision at 0.5, 1.0, and 2.0 cm from the root base. A total of ten embryos were used per excision treatment and the experiment was performed with three replications. The development of lateral roots and plant growth were then observed in each excision treatment at three time points, i.e., 1, 2, and 3 months after excision, in comparison with the control (samples with intact primary roots). The overall results showed that root excision treatments induced the development of lateral roots as branching roots appeared to come out from the excised region together with white air breathing roots seen at the upper portion of the primary roots near the stem base. Interestingly, we observed that shoots from the root-excised plants elongated considerably faster than the controls (Figure 1). A comparison of the lateral root number and shoot length between the plants in the three root-excision treatments and controls revealed that the plants in all root-excision treatments had a significantly higher number of lateral roots and higher shoot length than those in the controls (Figure 1a–c).

**Figure 2:**
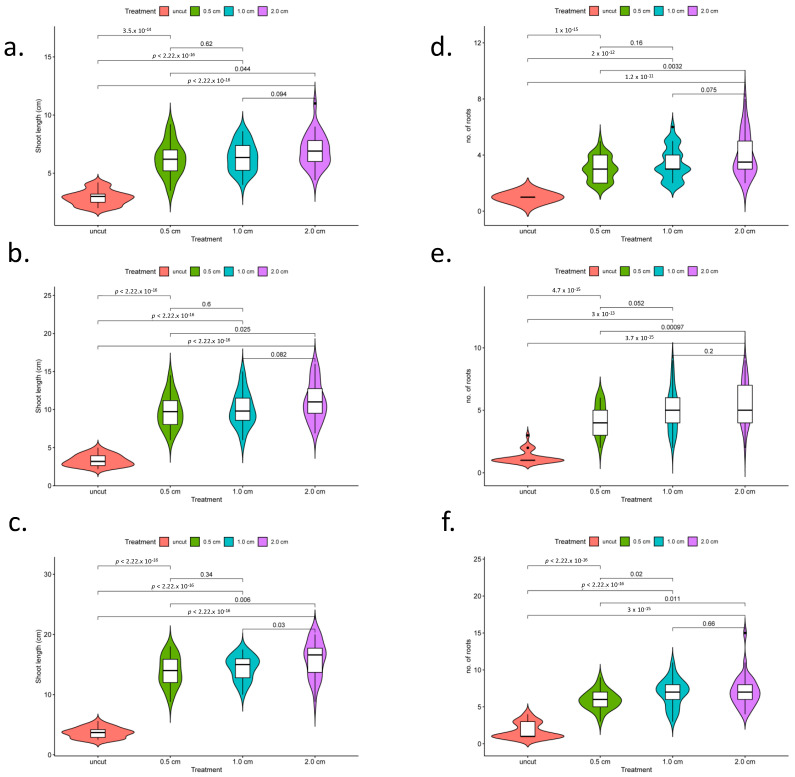
Comparisons of the shoot lengths and number of lateral roots in samples with different root excision patterns and controls (uncut) at three timepoints. Violin plot, box plots of shoot length at one month (**a**), two months (**b**), and three months after root cutting (**c**). Violin plot, box plots of the number of lateral roots at three timepoints, one month (**d**), two months (**e**), and three months (**f**) after root excision.

Moreover, we found that the number of lateral roots and shoot length were significantly higher in the treatment of root excision at 2.0 cm from the base, while these traits did not significantly differ between the treatments of root excision at 0.5 cm and 1.0 cm from base (Figure 2a–f). In addition, from our observations 3–4 months after root excision, all plants consistently grew well, with balanced growth of roots and shoot and up to 2–3 true leaves (Figure 1c). Lateral root function enhances the ability of the root system to acquire water and nutrients from the soil in conjunction with the growth of plant. Therefore, the plants with excised roots grew significantly faster and were stronger than those with intact roots. These results suggested that root excision impacted on the development of lateral roots, promoting plant growth.

### 2.2. RNA Sequencing and Transcriptome-Assembly Quality Statistics

A total of six RNA-seq libraries were derived from three samples of excised roots and another three samples of intact roots of makapuno coconut seedlings. The IIlumina sequencing runs generated RNA-seq reads ranging from 69.87 million to 90.45 million reads in the six libraries. We performed a de novo transcriptome assembly with Trinity using combined RNA-seq data (excised + intact root samples) to construct a unigene (transcript) reference for gene expression quantification and differential gene expression analysis. As a result, a total of 541,700 transcript contigs were assembled, with an N50 length of 1897 kb and an average length of 975.3 kb (Table 1). Among these, 215,347 contigs could be annotated by hitting reference genes (NCBI’s nr database).

### 2.3. Differential Gene Expression Analysis for Excised and Intact Roots

**Figure 3:**
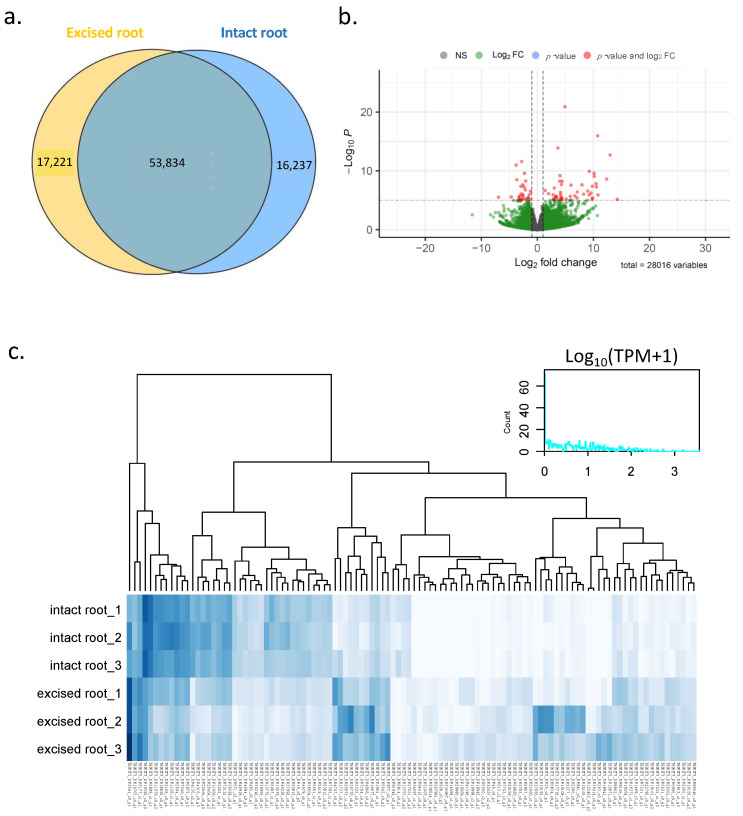
Gene expression in intact and excised roots. (**a**) Venn diagram of genes that were significant differentially expressed in intact and excised root samples. (**b**) Distribution and expression levels of differentially expressed genes (DEGs) of excised and intact roots; the *x*-axis represents the log_2_ (fold change) values under the mean normalized expression of all isoforms (*y*-axis). (**c**) Expression profiles retrieved from RNA-seq; the cluster displays expression patterns for a subset of genes with high log_2_ (fold change) values in comparisons between intact and excised root sampled with three replications.

In order to elucidate the molecular mechanism underlying lateral root development after primary root excision, we performed differential gene expression (DGE) analysis to identify genes that were differentially expressed between excised and intact root tissues. To obtain the expression of total transcripts in each of the six samples, we quantified the read abundance for each transcript in each sample by counting the RNA-seq reads that mapped to each transcript. We then filtered out lowly expressed transcripts to obtain only those transcripts that had a read abundance of greater than five reads in any sample. As a result, a total of 87,292 transcripts were selected. Of these, 53,834 transcripts were expressed together in both excised and intact root samples, 17,221 transcripts were expressed only in excised root samples, and 16,237 transcripts were expressed only in intact root samples (Figure 3). Based on DESeq2 results, a total of 512 transcripts were found to be differentially expressed when comparing between excised and intact roots (adjusted *p*-value: padj < 0.01) (Table S1). Of these, 271 differentially expressed genes (DE genes) were significantly upregulated and 241 DE genes were significantly downregulated in excised root samples.

### 2.4. Gene Annotation, Classification, and Pathway Analysis

**Figure 4:**
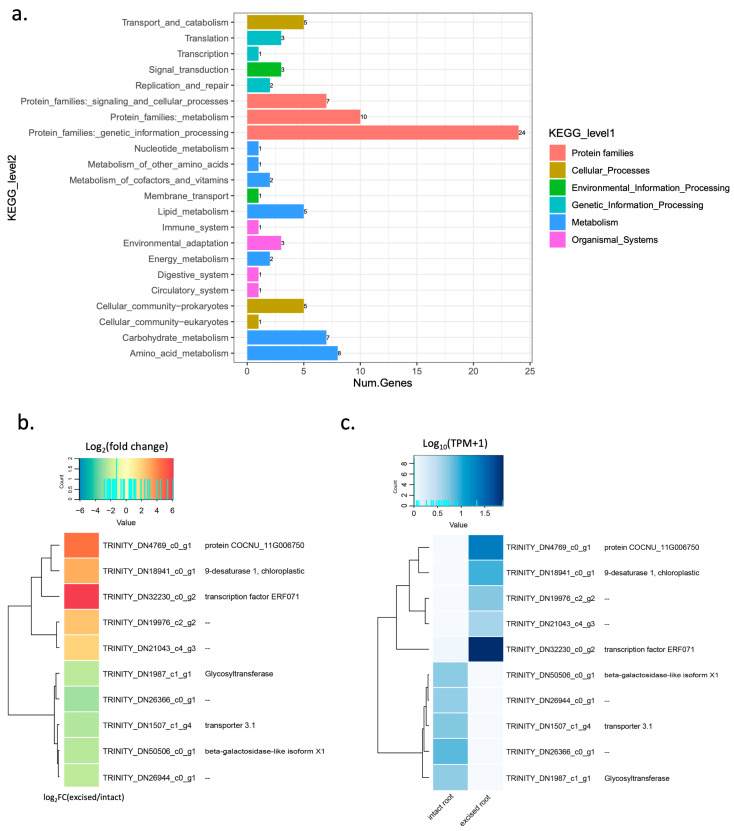
KEGG pathway enrichment of DE genes and the top twenty most highly expressed genes in comparison between intact and excised roots. (**a**) The KEGG pathways were summarized in six main categories. The *y*-axis indicated the name of the KEGG metabolic pathways. The *x*-axis indicated the percentage of the number of genes annotated under that pathway. (**b**) Hierarchical clustering heatmap of log_2_ (fold change) of the twenty most highly expressed transcripts. (**c**) Hierarchical clustering heatmap of log_10_TPM of the twenty most highly expressed transcripts.

We performed KEGG analysis to identify the biological functions and metabolic pathways of the DEGs involved in lateral root development. KEGG enrichment analysis revealed that the most enriched pathways were genetic information processing and metabolism (Figure 4a). Of 512 DE genes, 175 were annotated with a function (Table S1). We then selected the most highly expressed DE genes (TPM > 10) as candidate genes for further analysis (Table S2). A heat map of the expression of the 20 selected genes was plotted, comparing the intact and excised samples. As a result, we found that these 20 genes showed a differential expression pattern in intact and excised roots, as 15 of them were upregulated in excised roots, and the other 5 were upregulated in intact roots (down regulated in excised root). Among these genes, the ethylene-responsive element subfamily (ERF), *ERF071,* was the most highly expressed DE gene in the excised root (Figure 4b,c and Table S2). In addition to *ERF071*, *pyruvate*, *orthophosphate dikinase* (*PPDK*) was also upregulated in the excised root. The other 13 genes that were most highly expressed in the excised root were genes encoding uncharacterized protein or unannotated genes. Five genes were also found to be the most highly expressed genes in the intact root, but they were downregulated in the excised root. These included two-component response regulator *ARR17* and an uncharacterized *acetyltransferase*. The other three of the most highly expressed genes in the intact root have no annotated function. It has been reported that the *ERF071* gene in Arabidopsis plays an important role in root development via regulating root cell expansion [25]. This indicates that *ERF071* may play a similar role in coconut roots. Based on these results, we considered *ERF071* the most promising candidate gene involved in lateral root formation in coconuts.

### 2.5. Microscopic Observation of Lateral Root Formation

**Figure 5:**
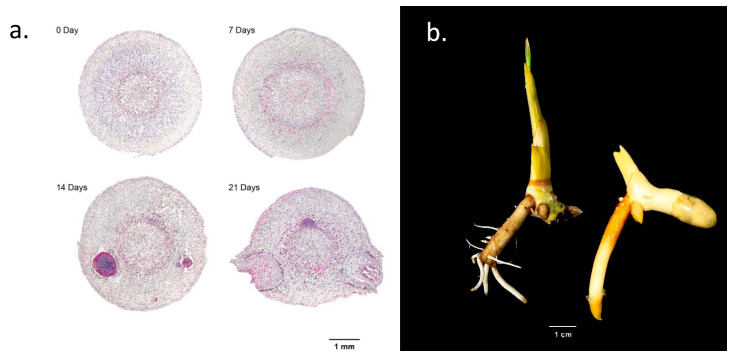
Lateral root development after excision was visible at 2 weeks after root excision. (**a**) Transverse section of the root showing lateral root formation on day 0, day 7, day 14, and day 21 after primary root excision. (**b**) A month-old makapuno seedling with lateral root growth around the excised area at the primary root that also showed induced shoot growth (left) compared to a makapuno seedling of the same age with an intact primary root that had no lateral root and much less shoot growth.

In order to understand the process of lateral root formation induced by the excision of the primary root, we took cross-sections of the primary root with a vibratome and observed the cellular tissue under a microscope. The cross-sections of the primary root were taken at one-week intervals, and four root sections were taken at day 0, day 7, day 14, and day 21, respectively. A series of microscopic images of root sections showed that, on day 0, only a normal structure of root cells was observed (Figure 5a). At seven days after root excision (day 7), cell movement was observed around the pericycle and endodermis, indicating the onset of lateral root formation. The onset of lateral root formation was clearly observed around the cortex area at 14 days after root excision, and at 21 days, lateral roots protruded to the outer surface of the cut edge (Figure 5a). The emergence of lateral roots was observed around the cut area of the primary root (Figure 5b).

### 2.6. Expression Analysis of ERF071 Using qRT-PCR

**Figure 6:**
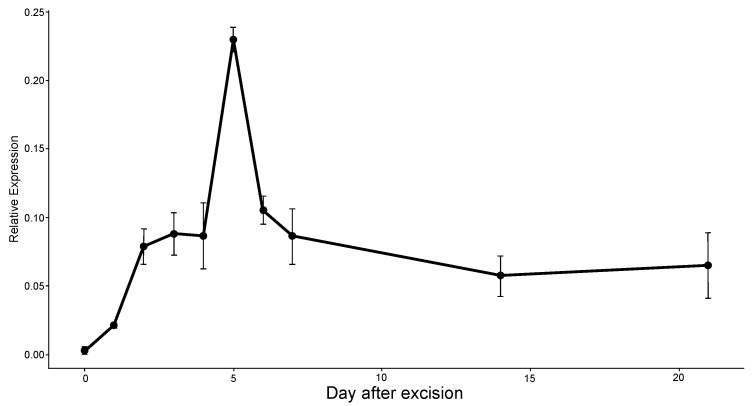
qRT-PCR analysis of *ERF071* in roots from makapuno at different time points after root excision. The relative expression of *ERF071* at each time point was calculated by comparison with the expression at day 0.

Gene expression analysis was performed using qRT-PCR for the candidate gene, *ERF071*, in order to verify its function in lateral root development after root excision. The level of relative expression was analyzed on each day of the first week, then 14 days and 21 days after root excision. The results showed that the expression level of *ERF071* was observed at a low level on day 0 and then gradually increased on later days (Figure 6). The highest expression level was observed on day 5 and it started to decrease on day 6 and 7. The expression level remained stable at two weeks and 3 weeks after root excision (Figure 6). The *ERF071* expression timing was consistent with lateral root formation, as lateral root initiation occurred between 7 days and two weeks after root excision (Figure 5). This suggests that *ERF071* may play a role in the early stage of lateral root initiation. Overall, our data suggested that root excision hypothetically contributes to the activation of *ERF071*, and this subsequently enables the formation of new lateral roots.

## 3. Discussion

In this study, we investigated the effects of primary root excision on promoting lateral root formation in makapuno coconut during embryo culture. The Y3 gelled medium used in this study was found to be suitable for makapuno embryo culture. Compared to the MS medium [26], which is widely used in tissue culture, the ammonium and nitrate nitrogen content in the Y3 medium is half as high, while the concentration of microelements such as iodine, copper, and cobalt is ten times higher. These changes may better reflect the conditions of a coastal soil, a favorable habitat for coconut germination [27]. It has been reported that culturing makapuno embryos by varying the sugar concentration, adding specific plant growth regulators such as NAA and BA to the Y3 medium, and solidifying the medium by adding agar resulted in an increase in plant survival rate in soil to 76% [28]. Our results showed that the combination of BA and NA, added to the Y3 gelled medium resulted in 80–100% germination after 2 months of culture. This could be the optimal combination of auxin and cytokinin ratio for mature embryo seeds to germinate.

The cultured embryos with excised roots had a higher number of lateral roots and better shoot growth. The results in the present study were similar to those previously reported in Arabidopsis showing that root excision promoted the regeneration of the root system by increasing the number and growth of both lateral roots and adventitious roots [13]. Wounding induces several local and systemic defense-related proteins and activation of hormones including ethylene [29]. Using a transcriptome analysis approach, we identified a large number of differentially expressed genes (DE genes) in excised and intact root samples. Additionally, based on the results of KEGG pathway enrichment analysis, we found that these DE genes belonged to several pathways, including genetic information processing and signaling pathways. Among the 20 most highly expressed genes (Table S2), *ERF071* was the most highly expressed gene in excised primary root. We considered *EFR071* as the most promising candidate gene associated with lateral root formation induced by root excision. In Arabidopsis, *ERF071* was also reported to play an important role in root development [25,30,31]. The ERF sub-family serves as important regulators in many biological and physiological processes, including response to wounding stresses [32]. Meanwhile, some of the ERF subfamily genes were reported to play roles in root initiation and the development of plants [33]. The AP2/ERF is a plant-specific superfamily of transcription factors (TFs) with 147 members in Arabidopsis [34] and 210 members in *Zea mays* [35]. However, the total number of this TF family in coconut has not been reported. ERF071 or HYPOXIA-RESPONSIVE ERF 2 (HRE2) belongs to the ERF-VII group of the AP2/ERF superfamily that the genes in this group have been implicated in the response to several stress factors [36]. The number of genes in this group varies from 3 in *Vitis vinifera* to 5 in Populus and 15 in *Oryza sativa* [37]. In addition to *ERF071*, *pyruvate*, *orthophosphate dikinase* (*PPDK*) was also found to be upregulated in excised primary root. PPDK is known to be a gene associated with C4 photosynthesis. Whether this gene is involved in lateral root development is not known. In this study we also found *ARR17* to be one of five most highly expressed genes in intact root. This gene was downregulated in the excised root. *ARR17* encodes a type-A response regulation involved in cytokinin hormone signaling [38]. Since cytokinin is mainly biosynthesized near the root transition zone [39], the primary root excision could affect cytokinin levels. Therefore, this might be the reason why *ARR17* was downregulated in the excised primary root.

Our observation of root sections showed an indication of cell movement at the pericycle near the endodermis seven days after root excision. Lateral roots normally arise primarily from the pericycle, a uniserate cylindrical layer of cells surrounding the central vascular cylinder of mature roots [40,41,42]. In Arabidospis, lateral root development initiation is indicated by pericycle cell division [22,24]. This suggests that the initiation of lateral root in makapuno coconut may have already started before the root section was observed. Several phases of pericycle cell divisions will create single layered primordia and, followed with subsequent periclinal divisions, will result in a dome-shaped lateral root primordia, which eventually emerges from the parental primary root [16,22,43]. When we observed root sections at day 14, lateral root had formed, and fully emerged lateral roots were seen at day 21. Lateral root primordia, therefore, can be assumed to start between 8 days to 14 days after root excision, e.g., 10 days (Figure S1), and fully emerge before 21 days. Furthermore, our qRT-PCR results showed the highest peak of *ERF071* gene expression at day 5 after root excision, which was moderately decreased at day 7. It can be assumed that *ERF071* activation may have happened at the early stage of lateral root initiation.

**Figure 7:**
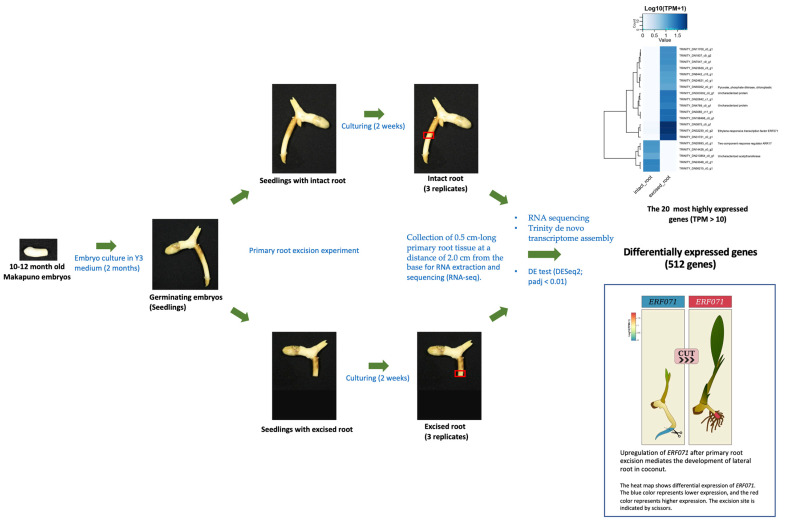
Schematic representation of the primary root excision experiment and transcriptome analysis showing the induction of *ERF071* after primary root excision mediating the development of lateral roots in coconut.

Several studies have shown that auxin plays an important role in lateral root (LR) formation [44,45]. During LR formation, auxin signals are transported to the protoxylem pericycle cells and auxin flux is mainly mediated by PIN-FORMED (PIN) family transporters which regulate auxin distribution in plant tissues [46]. It is widely known that auxin is a central regulator of LR formation in plants, but other hormones also positively or negatively affect LR formation at various stages. In this study, we identified the ethylene responsive factor, *ERF071*, which was activated as a response to the root excision of makapuno coconut (Figure 7). Based on the results of the present study, we hypothesized that *ERF071* may contribute to LR initiation. To the best of our knowledge, this is the first report on genes associated with lateral formation induced by primary root excision in coconuts. However, further study is necessary to confirm the function of *ERF071* in lateral root development in coconuts.

## 4. Materials and Methods

### 4.1. Plant Materials

Matured nuts (11–12 months old) of makapuno coconut were collected from Kasetsart University Coconut Biobank, Thailand. The fruit nuts were husked, the hard endocarp/shell were removed, and the portion of the kernels that enclosed the zygotic embryos were carefully extracted with care so as to not damage the embryos. These materials were kept in clean plastic bags and stored in cold conditions. The samples were disinfected in a laminar air flow chamber by rinsing in 70% ethanol for 30 sec, followed by immersion in 20% (*v*/*v*) commercial bleach (2.0–2.5% active chlorine) for 20 min. The samples were then rinsed in sterile distilled water and the embryos were extracted.

### 4.2. Embryo Culture Growth Conditions and Root Excision Treatments

For the culture medium, we used our developed medium for the whole culture process; Y3 medium [47] supplemented with BA and NAA. After adding commercial sugar 50 g/L to the medium, the pH of the medium was adjusted to 5.75. Then, gellan gum 3 g/L and activated charcoal 0.5 mg/L were mixed with the media solution and autoclaved for 15 min at 121 °C. After zygotic embryos were aseptically extracted, they were inoculated in bottles (30 mL) of culture medium. The initial cultures were incubated at 25 ± 2 °C in dark conditions for 2 months until almost all embryos were germinated with one primary root. Afterwards, they were transferred to culture shelves at the same temperature, under cool-white fluorescent lamps (50–60 µmol m^−2^ s^−1^) for a 16 h photoperiod. After 5 days under the light, the germinated embryos were collected for the root excision treatment, and their primary roots were excised in three patterns, i.e., excisions at 0.5 cm, 1.0 cm, and 2.0 cm from the root base. Subculture in fresh nutrient media was undertaken at four-week intervals during the culture period. The experiment was a complete randomized design (CRD) with three excision treatments and a control (intact root). Each treatment consisted of 20 embryos and was replicated three times. The data on shoot length (cm) and root number were taken at 1, 2, and 3 months after root excision.

### 4.3. RNA Extraction, RNA Sequencing (RNA-seq), and De Novo Transcriptome Assembly

Intact and excised root samples were collected two weeks after primary root excision and used to generate RNA-seq data. For the excised root samples, 0.5 cm root tissues were collected from the edge, including the previously excised area. For the intact root samples, the primary root was first cut at a distance of 2.0 cm from the base and then the 0.5 cm root tissue in the cut area was collected, as for the excised root samples. A total of six root samples from six embryos were collected, with the first three samples representing three replicates of the intact root control and the other three samples representing the excised root treatment. Total RNA was extracted from the six root samples using the RNeasy Plant Mini Kit (QIAGEN, Redwood City, CA, USA). The quality of the total RNA was assessed using a NanoDrop 8000 (Thermo Fisher Scientific Inc., Waltham, MA, USA). The extracted total RNA was stored at −80 °C before being sent for sequencing. RNA-seq library preparation and Illumina sequencing were carried out on an Illumina HiSeq 2500 PE150 platform (Illumina, San Diego, CA, USA) at Novogene Bioinformatics Institute (Beijing, China). The RNA-seq reads from all six libraries were pooled and used for de novo assembly of the transcriptome using Trinity assembler (v2.5), following previously reported approaches [48]. The Trinity workflow can be found at: http://trinityrnaseq.github.io (accessed on 9 October 2022).

### 4.4. Gene Annotation, Classification, and Pathway Analysis

The de novo transcriptome assembly was annotated against Nr (NCBI nonredundant protein sequences), Swiss-Prot, and KEGG (Kyoto Encyclopedia of Genes and Genomes) using the DIAMOND (BLASTX compatible aligner) program [49]. Functional and pathway enrichment analysis for differentially expressed genes were performed based on the KEGG pathway database [50].

### 4.5. Read Abundance Quantification and Differential Gene Expression Analysis

The gene expression levels of each gene in each sample were estimated by mapping the clean RNA-seq reads against the whole set of assembled contigs using Salmon [51]. Transcripts per millions mapped reads (TPM) was also calculated using the same software. Differential gene expression (DGE) analysis was performed using the DESeq2 package version 1.34.0 [52] by comparing three replicates of intact root samples and three replicates of excised root samples. Parameters were set based on the user manual of the software package. The *DESeq* function was used to perform the differential analysis, and the results were generated using the *results* function. The significant threshold for determination of the differentially expressed (DE) genes was a false discovery rate (FDR or adjusted *p*-value: padj), <0.01. The most highly expressed DE genes were selected using the threshold of an average TPM > 10 in each group of root samples.

### 4.6. Microscopic Observation

To study the lateral root development after root excision, a 1 cm section from the excised edge of the root was collected at 0, 7, 14, and 21 days after primary root excision. For the observation, thin sections of 20–50 micron in thickness were acquired using Vibratome sectioning. Sectioned samples were transferred to distilled water in a Petri dish which contained 0.1% solution (*w*/*v*) of Toluidine Blue O (C.I. 52040, Sigma). Specifically, intact thin sections were transferred into a clean Petri dish and one drop of Toluidine Blue O solution was placed directly onto the thin section in the Petri dish for 20 s. Tissues were then rinsed three times with distilled water and removed from the Petri dish. The samples were mounted on a microscopic slide with one drop of water and covered with a cover slip. Examination and imaging were carried out under the stereo microscope (LEICA EZ4W), which was integrated with a 5-megapixel camera.

### 4.7. Expression Analysis by Quantitative Reverse Transcription PCR (qRT-PCR)

The qRT-PCRs were conducted using iScript One-Step RT-PCR reagent with SYBR Green (Bio-Rad, USA), using 1 ng of total RNA as the template. Ubiquitin-conjugating enzyme E2 10 (UBC 10) forward primer was included as an internal reference gene to normalize the variations of input total cDNA template. The gene-specific primers were forward 5′ GTT CAT CCC TCG CCG GAA AA 3′ and reverse 5′ GGC TCG GGA GTC TTA GGG AA 3′, and the ubiquitin primers were forward 5′ CAG GAC CCG TGG CAG AAG A 3′ and reverse 5′ TAG AAA GAC ACC CCC AGC ATA AG 3′. The relative gene expression was analyzed by Bio-Rad CFX Manager analysis software (BioRad, Hercules, CA, USA) in order to compare fold changes in expression relative to ubiquitin as a control.

## 5. Conclusions

This study provided insight into the mechanism and candidate genes involved in the induction of lateral root development in coconuts, which may be useful for the future breeding and mass propagation of makapuno coconuts using tissue culture. The candidate gene *ERF071* was reported as a regulator for lateral root development in coconuts for the first time.

## Figures and Tables

**Table 1:** De novo transcriptome assembly statistics.

Statistics	Root (Excised + Intact)
Number of total contigs	541,700
Number of contigs hit reference genes	215,347
Total nucleotides (bp)	801,856,735
N50 length (bp)	1897
Average contig length (bp)	975.30
Min. contig length (bp)	178
Max. contig length (bp)	21,353

## Data Availability

The RNA-seq data described herein have been deposited in the NCBI repository, accession number PRJNA893805.

## Supplementary Materials

The following supporting information can be downloaded at: https://www.mdpi.com/article/10.3390/plants12010105/s1, Table S1: Differently expressed genes compared between excised and intact root samples; Table S2: The 20 most highly expressed DE genes; Figure S1: Transverse section of root showing lateral root formation at day 10 after primary root excision. Lateral root primordia are indicated by a yellow arrow.
