# Supplementary material for: Primary Root Excision Induces ERF071, Which Mediates the Development of Lateral Roots in Makapuno Coconut (Cocos nucifera)
Source: Plants (Basel). 2022 Dec 26;12(1):105. doi: 10.3390/plants12010105 (PMC9823405; doi:10.3390/plants12010105)
Supplement: Supplementary file 1 [file plants-12-00105-s001.zip › Figure S1 - cross section 10 days.pdf]

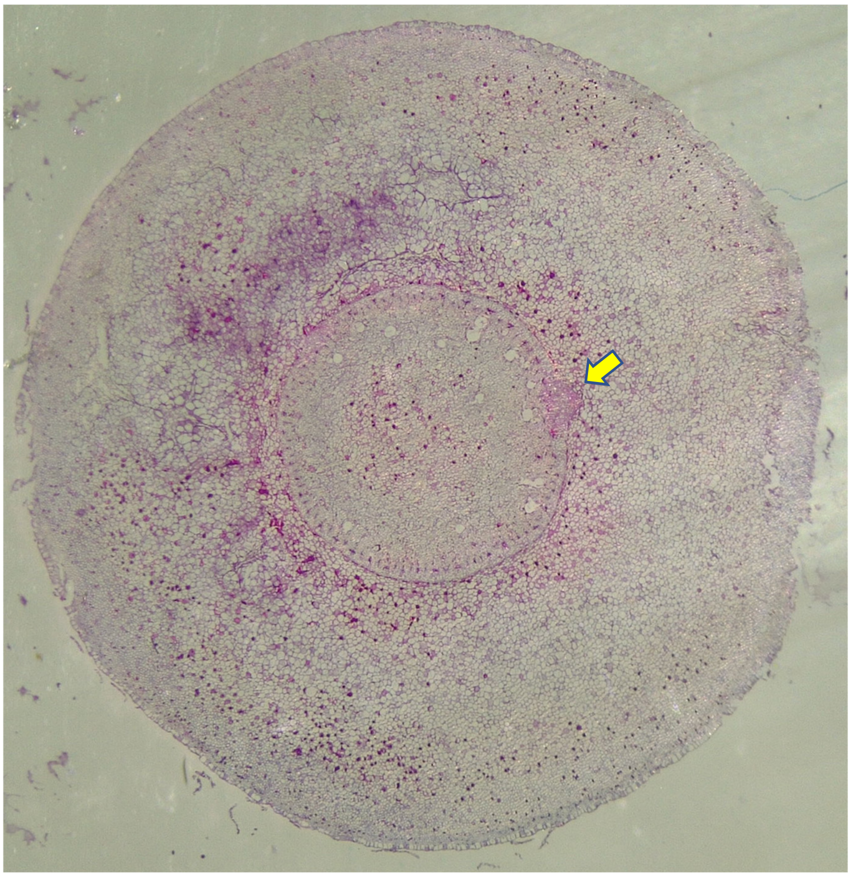

Figure S1 Transverse section of root showing lateral root formation at day 10 after primary root excision. Lateral root primordia are indicated by a yellow arrow.
